# Co-Operative Design of a Coach Dashboard for Training Monitoring and Feedback

**DOI:** 10.3390/s22239073

**Published:** 2022-11-23

**Authors:** Jos Goudsmit, Ruby T. A. Otter, Inge Stoter, Berry van Holland, Stephan van der Zwaard, Johan de Jong, Steven Vos

**Affiliations:** 1 Department of Industrial Design, Eindhoven University of Technology, 5612 AJ Eindhoven, The Netherlands; 2 School of Sport Studies, Fontys University of Applied Sciences, 5644 HZ Eindhoven, The Netherlands; 3 School of Sport Studies, Hanze University of Applied Sciences, 9747 AS Groningen, The Netherlands; 4 Department of Biomedical Sciences of Cells & Systems, Section Anatomy & Medical Physiology, University Medical Center Groningen, University of Groningen, 9713 GZ Groningen, The Netherlands; 5 Innovationlab Thialf, 8443 DA Heerenveen, The Netherlands; 6 Leiden Institute of Advanced Computer Science, Universiteit Leiden, 2333 CA Leiden, The Netherlands; 7 Human Movement Sciences, University Medical Center Groningen, University of Groningen, 9713 GZ Groningen, The Netherlands

**Keywords:** dashboard, training load, coach, feedback, athlete development, performance, overreaching, overtraining

## Abstract

Athlete development depends on many factors that need to be balanced by the coach. The amount of data collected grows with the development of sensor technology. To make data-informed decisions for training prescription of their athletes, coaches could be supported by feedback through a coach dashboard. The aim of this paper is to describe the design of a coach dashboard based on scientific knowledge, user requirements, and (sensor) data to support decision making of coaches for athlete development in cyclic sports. The design process involved collaboration with coaches, embedded scientists, researchers, and IT professionals. A classic design thinking process was used to structure the research activities in five phases: empathise, define, ideate, prototype, and test phases. To understand the user requirements of coaches, a survey (*n* = 38), interviews (*n* = 8) and focus-group sessions (*n* = 4) were held. Design principles were adopted into mock-ups, prototypes, and the final coach dashboard. Designing a coach dashboard using the co-operative research design helped to gain deep insights into the specific user requirements of coaches in their daily training practice. Integrating these requirements, scientific knowledge, and functionalities in the final coach dashboard allows the coach to make data-informed decisions on training prescription and optimise athlete development.

## 1. Introduction

In their ambition to become elite athletes, the development of young athletes is crucial and depends on many factors that must be balanced [1]. These factors are categorised as training load, physical performance, mental health, and daily life (e.g., school and social) factors [2]. Coaches need to manage these factors to guide young athletes in becoming elite athletes and prevent them for non-functional overreaching, overtraining, and injuries [2,3]. In order to make appropriate decisions for athlete development, a considerable amount of training data (e.g., heart rate, velocity) and recovery data (e.g., sleep, muscle soreness, perceived exertion) is collected [4,5]. However, collecting, processing, and interpreting all these data can be difficult and time-consuming for the coach, even though this has potential benefits for the coach. Relevant feedback and insights can help coaches to use data more efficiently and have better control over designing training programmes and their effect on the athletes.

Cyclic sports (e.g., speed skating, cycling, and running) are characterised by repetitive activities and have a major focus on physiological aspects (e.g., exerted power, heart rate, oxygen uptake, and so on) [4]. Therefore, these types of sports are of especial interest to dashboard developers because monitoring training load and physical performance can be measured and influenced well by different available technologies. Quantification of physiological aspects can be performed using internal and external training-load measures and monitoring [6]. External load refers to the output performed by the athlete and internal load is the athlete’s mental and physiological response to that output [6]. Many wearable sensors are available to measure the external training load [7,8]. For speed skating, measuring velocity by lap times or even parts of laps is a relevant and feasible marker for external load [9]. Internal load parameters are measured continuously during training and exercise using sensors and can be complex to analyse. To illustrate, analysing and interpreting heart rate sensor data can be performed in all kind of manners, such as defining maximal heart rate, average heart rate, Training Impulse (TRIMP) scores, time in heart rate zones, and so on [10]. Insights from this internal load should be compared to the external load measurements, which have the same or even more analysis and interpretation options. In addition to these objective load parameters, there is also the perceived internal load of the athlete. In particular, athletes’ perceived training satisfaction and training load using Rating of Perceived Exertion (RPE) are relevant and frequently used subjective measures to assess the impact of training on the athlete [11,12,13]. Choosing appropriate analyses, interpretation, and decision making to adapt upcoming training programmes and sessions challenges the skills, knowledge, and time of the coach [14,15]. This complex challenge leads to the problem at hand: how can the coach be informed about training load and subjective measures to gain insights into the impact of training on the athlete and adapt their training programmes accordingly?

Coaches who cannot call on experts could be supported by feedback through a coach dashboard to gain insights on training load and subjective measures in a simple, feasible, and informative way [16,17]. Data science and business intelligence facilitate the development of interactive dashboards that help users to analyse and interpret large quantities of data [18]. Equipping the coach with a coach dashboard would enable informed, science-based, and personalised decision making for training optimisation. Successful implementation and acceptance of technological innovations, such as a coach dashboard, by coaches is only possible when coaches see the added value to their work and when the dashboard fits into the existing work processes and routines [19,20]. Therefore, a co-creation process between science and practice is needed for careful implementation and involvement of coaches during the development process of such a coach dashboard [20]. We used a co-operative research design and involved coaches, the target users of the coach dashboard, in the design process to increase the likelihood of adoption and implementation of the coach dashboard in practice [21,22].

In this descriptive research paper, we describe the process of designing a coach dashboard based on scientific knowledge, user requirements, and (sensor) data that supports coaches of athletes in decision making about training prescription for athlete development in cyclic sports. The development process involved collaboration with coaches, embedded scientists (i.e., sport scientists in the field), researchers, and IT professionals. First, we identified user requirements and scientific knowledge available on training load that were translated into design principles for a coach dashboard. Then, mock-ups of the dashboard were designed, and subsequently a prototype application was designed in several iterations. Finally, the minimum viable product dashboard was examined, including how it meets the needs of coaches, and the bottlenecks experienced by coaches.

## 2. Materials and Methods

In designing the dashboard, coaches were involved in the co-operative research design. The co-operative research design was part of the so-called Coach in Control project, involving many stakeholders. Stakeholders involved, besides coaches, were software engineers and data scientists from the Sport Data Valley platform [23] who build and host the coach dashboard, innovation lab manager, and human-movement scientists. Stakeholders helping to implement the dashboard in the coaches’ daily practice were national sports associations (e.g., NOC*NSF and KNSB) and elite sports networks.

We used a classic design thinking process [24,25] to structure our research activities in five phases: empathise, define, ideate, prototype, and test phases.

Target users for the dashboard were coaches working with talented speedskaters and runners. These coaches are best described as coaches in individual sports, coaching a performance-driven group of young athletes. The athletes have 5 to 8 training sessions during the week, so training load needs to be closely monitored and aligned with the athletes’ development. Additional support from an embedded or data scientist is not available for these groups and their coaches. Coaches with performance-driven groups of athletes are mainly educated in educational programmes delivered by sports associations at EQF level 4 [26] and they have ambitions to develop their knowledge and skills in coaching athletes based on monitoring training.

### 2.1. Empathise Phase—Importance, Needs, and Bottlenecks

A survey was constructed based on a narrative literature review on monitoring aspects during and around training, overreaching, and overtraining [5,7,12]. The aim of the survey was to empathise with the importance, needs, and bottlenecks experienced by coaches in collecting, analysing, and interpreting training and health data. We identified 48 factors that can be used to quantify training load, physical performance, mental health, and daily life. The importance and use of items were collected using a 5-point Likert scale [27]. Items were categorised with respect to data collection before, during, and after training sessions, performance test data, and competition result data. The bottlenecks and needs were further questioned in a more practical sense. For example, the time needed for training monitoring, costs, existing dashboards, tools, support coaches already receive, and so on. All survey items were validated in consult with six experts, namely, an embedded scientist working with elite-level athletes, a sports innovation lab manager, an elite coaching director, an innovation performance manager, and two human-movement scientists.

The survey was sent to 200 selected coaches EQF level 4 [26] by the national sport association (NOC*NSF) and 6 specific national sports associations (speed skating, running, rowing, swimming, cycling, and triathlon). Coaches working in these sports were selected for the nature of the sport as a cyclic sport, because in these types of sports, monitoring training and health is already adopted [4]. After two weeks, the survey was further spread via social media (Twitter and LinkedIn) and regular reminders were sent within the next six weeks. Results were analysed using non-parametric statistical testing (Friedman) for relevant constructs and questions. A *p*-value smaller than 0.05 was considered as statistically significant.

For more in-depth insights into the survey results, eight online interviews were conducted. Interviewed coaches were elite-level coaches already using training and health monitoring in speed skating, cycling, running, rowing, and swimming. The topics addressed were the same as the constructs in the survey. More focus was put on the bottlenecks, specific monitoring measures, and in-practice use of monitoring systems that these coaches experienced and used. Thematic analysis was performed using transcription of the interviews. Themes were adopted from the survey data (e.g., monitoring before, during, after training, performance tests, and competition) and additional themes were adopted from the interviews themselves.

### 2.2. Define Phase—Coach Dashboard Design Principles

The results of the survey and interviews were used as input for four focus-group sessions with four participants each which aimed to determine the design principles for the coach dashboard. Selected participants formed an interdisciplinary group of one talent coach, one elite coach, one human-movement scientist, and one IT specialist. To structure and facilitate the discussions with these four participants, COMMONS [28] was used. COMMONS is a research-based tangible board game developed to facilitate interdisciplinary decision making and alignment of priorities in a design process. Statements were discussed, 30 in total, in relation to a given use case. Discussions were organised in 3-min rounds during the game. The used case (Appendix A) and 30 statements (Appendix B) were constructed from the survey and interview results. Data were analysed using ranking procedures to prioritise the discussed statements. Ranking procedures were based on the scores for each statement at the end of the focus-group session. These rankings were supplemented with given reasons during the discussion. All sessions started with providing consent by the participants. Sessions were held online using MS Teams due to COVID-19 measures. Hence, COMMONS was adjusted to a digital format (Appendix C). All discussions were screen- and audio-recorded and analysed by thematic analysis as well.

### 2.3. Ideate Phase—Mock-Up Design

With the results of the define phase, two iterations of mock-ups were drawn. The development team included a human-movement scientist, data scientist, IT specialist, and an innovation lab manager. The first iteration was discussed in focus-group sessions with three coaches, and the second iteration with five coaches, working with elite-level and development-level athletes. The discussion after displaying the mock-up was structured using the concepts functionalities, content, design, overview, simplicity, and insights. These concepts were constructed based on thematic analyses of the focus-group sessions in the define phase. Participants were asked to score every concept using a 5-point scale after discussing the aspects. Thematic analysis was performed based on the transcription of the discussions with coaches.

### 2.4. Prototype Phase—Minimum Viable Product Dashboard

Prototyping started based on the final mock-up and already-existing functionalities and infrastructure related to monitoring of training load and well-being within the Sport Data Valley platform [23]. This online platform provides a solution that enables researchers and coaches to easily capture and analyse data, but with data privacy ensured. The following relevant infrastructure of the platform was already in use: (i) daily questionnaires for athletes to collect health and well-being status (based on [29,30,31]); (ii) training log questionnaire to collect data on the perceived training load (based on [13]), (iii) training-sensor data integration with wearable systems such as Polar, Garmin, Strava, and Fitbit, (iv) functionalities to connect and share data between athletes and coach, and (v) dashboards displaying questionnaire and training activity data. A dashboard informing the coach about data for an athlete, or a group of athletes, was, however, not present.

The Agile development method with a derivate of the Agile Scrum method [32] was used during this prototype phase. The developer (scrum team) and one of the human-movement scientists (product owner) collaborated in this phase building the minimum viable product, i.e., Coach in Control dashboard in weekly sprints. After four weekly sprints, the most recent prototype of the coach dashboard was showcased to a team of three human-movement scientists and three elite coaches. The coaches were selected for their experience using training-load monitoring systems. Group discussions were recorded to perform thematical analysis of the discussed topics.

### 2.5. Test Phase—Practical Use of the Coach in Control Dashboard

During the test phase, the minimum viable product dashboard was used and implemented by four coaches in their team and training situation. Coaches were selected based on their former experience with a coach dashboard system and their shown motivation to use the developed Coach in Control dashboard. Two coaches had a small number (1 to 4) of their athletes use the platform to provide the necessary input for the Coach in Control dashboard. Two coaches who were already using other functionalities within the Sport Data Valley platform [23] implemented their complete group of athletes (12 and 14 athletes). After 6–8 weeks of using the dashboard, 30 to 45 min structured interviews were held with the coaches. Concepts as used in preceding phases (functionalities, content, design, overview, simplicity, and insights) were discussed in these interviews for the two implemented dashboard pages. Interviews were performed to collect specific and practical qualitative data to improve the dashboard for the training situation of coaches. Thematical analysis was used to analyse the interviews. In this phase, we only collected qualitative data to evaluate the dashboard.

## 3. Results

### 3.1. Empathise Phase—Importance, Needs, and Bottlenecks

Thirty-eight coaches returned the survey and interviews were conducted with eight coaches. The importance and use-in-practice of monitoring in training, testing, and competition monitoring is displayed in Table 1. There were no differences (*p* = 0.307) in the importance of collecting training data (4.0), performance testing data (3.9), and competition data (3.8). Reasons for collecting and using data were to implement adaptations of the individual training load (4.1) and to follow the athletes’ development (4.1). Monitoring before training is considered the most important (3.9; *p* < 0.001 to all other “Importance” categories), while monitoring during training is found to be least important (3.3; *p* < 0.001 to *p* = 0.044 to all other “Importance” categories). All monitoring categories were at least classified as reasonably important or higher. How coaches use monitoring in their practice was different from what they classified as most important. For example, monitoring before training comes as third most-used, while it was also classified as most important. Statistical analysis showed that the importance of all training monitoring and competition monitoring data was different from the use of monitoring in practice (before training *p* = 0.016; during training *p* = 0.016; after training *p* < 0.001; competition *p* < 0.001). These differences show that coaches experience barriers to monitor in practice what they classify as important. Performance testing showed no difference between importance and use (*p* = 0.622). The interviews showed similar results and learned that coaches use monitoring before training to make last-minute adjustments for the upcoming session. “First thing I do in the morning is check if all athletes filled in their daily questionnaire and if any injuries or sickness have been reported” (C1). Coaches also talk to athletes to find out how they are feeling before training without registration of the information athletes provide during those conversations. “When an athlete reports an injury in the morning, I check what still is possible for the upcoming training session” (C1).

Available measures were ranked to identify the most important measures in monitoring (see Table 2 for a complete list). Measures seen as most important to collect before training were injuries (4.6), fatigue (4.4), and recovery (4.4). During training, the technical execution of the athletes (4.4) is most important, followed by heart rate (3.8) and power (3.7). After training, coaches found the experienced fun (4.3) most important, followed by perceived load (3.8) and training duration (3.6). Interview results showed that several combinations of measures were of main interest for coaches. For example, velocity versus heart rate during training. “Within all monitoring data I start with looking at figure displaying heart rate vs. skating velocity because I want to see the reaction of the body to the velocity the speed skater develops.” (C2). In addition, technical execution of a movement skill was also classified as important. However, in practice, this was only observed by the coach, with no additional record or registration in monitoring systems. “I observe the technique and provide feedback to the athlete based on my observation. I do not keep records on this observation, I just know how the athlete moves because I see it almost every day” (C1). Therefore, technique is considered as important, coaches provide feedback on it in almost every training session, but to keep track of the improvements, coaches rely on their memory.

**Figure 1:**
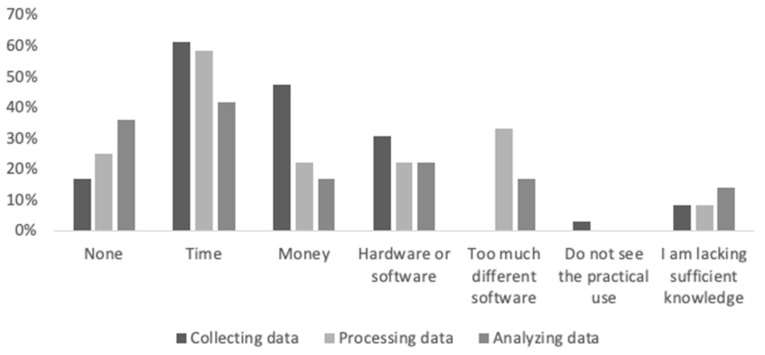
Perceived bottlenecks by coaches in collecting, processing, and analysing training monitoring measures. Coaches were able to choose multiple bottlenecks.

Most frequently experienced bottlenecks (see Figure 1) are not having enough time for the coach to collect data (61%), process data (58%), and analyse data (42%). Insufficient available money, hardware, and software are also mentioned by one-third of the respondents. Coaches experience most bottlenecks in collecting data, followed by processing data and analysing data. Coaches reported using their own-built monitoring system (60%) and consumer-available systems such as TrainingPeaks, Garmin Connect, Polar Flow, and Strava. Most coaches (60%) used two to five different systems to monitor their athletes. Interviewed coaches showed their monitoring system during the interviews and demonstrated how they worked with it, which gave insight into the bottlenecks addressed. A specific example addressed was the large number of figures and feedback provided by the used system (e.g., TrainingPeaks) leaving the coach with the decision of which feedback to use for training design. “TrainingPeaks shows a lot of information, but I only look at a couple of the figures that are presented. I gain the most information out of the daily workload figure.” (C3).

The survey results provided the initial coach dashboard design principles. Functionalities and feedback provided by a dashboard are rated as equally important (3.9 over 3.7; *p* = 0.063) by the respondents of the survey. Both are classified as ‘important’ with ~4 out of 5 points. The highest-ranked functionalities by coaches are “Select myself which measures to see”, “Short navigation (few mouse clicks) from an overview panel”, and “Being able to design my own overview page”. The most important feedback design principles were “Load of the individual athlete per week”, “Detailed feedback on individual data”, and “Load of the individual athlete per session” (all functionalities and feedback principles are displayed in Table 3).

To give a first estimation on the time that the coach and the athletes want to invest for monitoring, respondents had to give the time they actually want to spend on monitoring. Most coaches would like to spend less than 10 min before training (66% of the coaches), less than 5 min during training (54% of the coaches), and 6 to 20 min after training (63% of the coaches). Athletes should be able to enter their data in no more than 10 min a day, according to 80% of the coaches. Interviewed coaches provided a nuanced picture of the invested time. When a monitoring system or coach dashboard provides meaningful feedback, it could save time compared to the present workflow used in practice. Moreover, when a coach dashboard provides relevant, important, and valued insights, then the invested time is well-spent. “On Saturdays I spend a couple of hours now to go through the monitored data and training logs of the athletes. Most of the time I spend on getting all the needed information so only little time is left for getting full insights of all monitored data.” (C4).

### 3.2. Define Phase—Coach Dashboard Design Principles

The survey results and interviews were used to develop a use case and 30 statements to facilitate the discussions in the focus groups. In the use case, the most important monitoring data that should be collected and displayed in the coach dashboard and characteristics of the coach that will be using the coach dashboard were described.

Displayed results in Table 4 are ranked statements averaged over the four focus-group sessions, with four participants each. The highest-ranked statement was “In a maximum of 20 min the coach should be able to use all individual training monitoring for the next training session”, followed by the statement “When athletes put in the system that they are injured there will always follow a warning” and “With large differences within an individual the dashboard sends active warnings”. Reasons given during the discussions of the statements were considered in defining the design principles. According to the participants, feedback should be based on a combination of external load, internal load, and subjective data. All relevant feedback should be based on the individual athlete’s data and give insight into the performance development of the athletes. Functional aspects such as time, warnings, combining existing systems, and flexibility are the highest-ranked items according to the focus-group sessions. Coaches want to spend as little time as possible in the practical use of a dashboard while gaining the most insightful feedback out of it. Provided feedback from a dashboard should help the coach in decision making by providing data without prescribing what to do. In this way, the coach can use its expertise to make data-informed decisions for training.

Concluding this define phase, measures and monitoring data to collect and functionalities of a coach dashboard are defined. The overview part of the dashboard should provide information and toggle on and off warning functionality for well-being, health, and training-load basics, displaying easy-to-interpret measures. Warnings should be based on relevant deviations from the individual athletes’ historical data. Feedback on training sessions and training-load data should have detailed session data such as time in heart rate zones, several average scores, and practical information such as training type, time, and duration. Furthermore, the intended training should be compared with the experienced and measured data during and after training by the athlete. In this way, the coach learns how to better adapt the design and objectives of his training sessions to the perceived load of the individual athletes. Training-load data should be combined over weekly periods and provided over a long period of time that the coach can choose, up to years back.

### 3.3. Ideate Phase—Mock-Up Design

**Figure 2:**
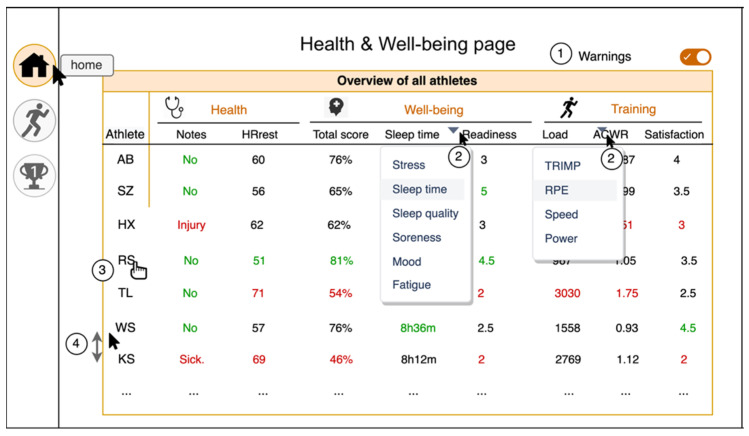
Display of the second iteration mock-up of the coach dashboard. Health and Well-being page where all athletes’ daily questionnaire data are displayed in overview. The numbers in the Figure are explained as follows: 1, Toggle warnings ON/OFF to display green (positive), black (average), and red (potential risk) colours in the overview when ON and all black when OFF; 2, Drop-down functionalities in well-being data and training data to specify by the coach which data should be displayed; 3, Clicking the initials of the athlete leads to the Training and Trend page of that athlete; 4, The order of athletes can be changed by clicking and dragging the athletes’ initials.

**Figure 3:**
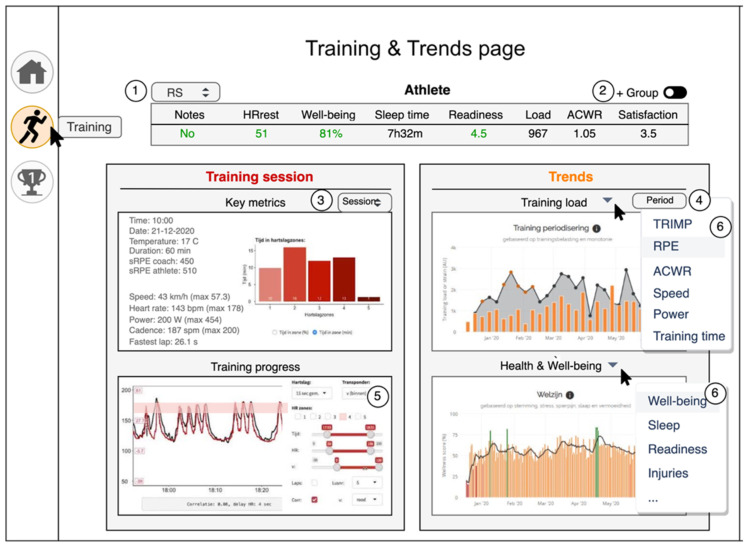
Display of the second iteration mock-up of the coach dashboard. The Training and Trends page where training-load data are displayed for the chosen athlete. The left panel shows data from a single activity. The right panel shows weekly trends of training load and well-being. The numbers in the Figure are explained as follows: 1, Initials of the chosen athlete; 2, Toggle to display the individual data in relation to the group average data; 3, Choose the training session to be displayed; 4, Choose which period should be displayed in the Trends panel; 5, Choose which data should be displayed in the training timeline figure and if heart rate zones should be displayed in the training timeline figure; 6, The coach can choose the data that are displayed using the drop-down functionality in the Training-load and Health and Well-being figures in the Trend panel.

The ideate phase took 6 weeks and resulted in a mock-up of the coach dashboard. Based on the results of the define phase, a two-page dashboard was drawn. The first page is a Health and Well-being page (Figure 2), which provides an overview of the athletes to the coach on daily reported health (injury, sickness), well-being (e.g., sleep, mood, stress, soreness), and readiness for training. The second page is a Training and Trends page (Figure 3), which displays training measures from the chosen training and trends over time for training load and well-being of the selected athlete.

The concepts, functionalities, content, design, overview, simplicity, and insights were tested during interviews with coaches. Interviews started with a showcase and explanation of the mock-up and functionalities, followed by discussion of the mock-up. Five participants scored the second mock-up for both the Health and Well-being page and the Training and Trends page. Functionalities (4.0), content (4.2), and insights (3.9) scored highest for both pages, while simplicity scored lowest (See Table 5).

### 3.4. Prototype Phase—Minimum Viable Product Dashboard

**Figure 4:**
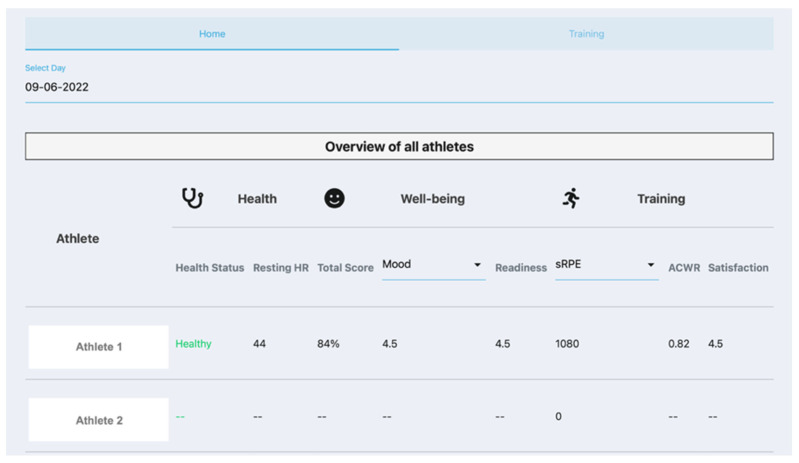
Display of the minimum viable product: Coach in Control dashboard. Heath and Well-being page overview. The Coach in Control dashboard is displayed as integrated in the Sport Data Valley platform [23]. Copyright 2022 Sport Data Valley.

**Figure 5:**
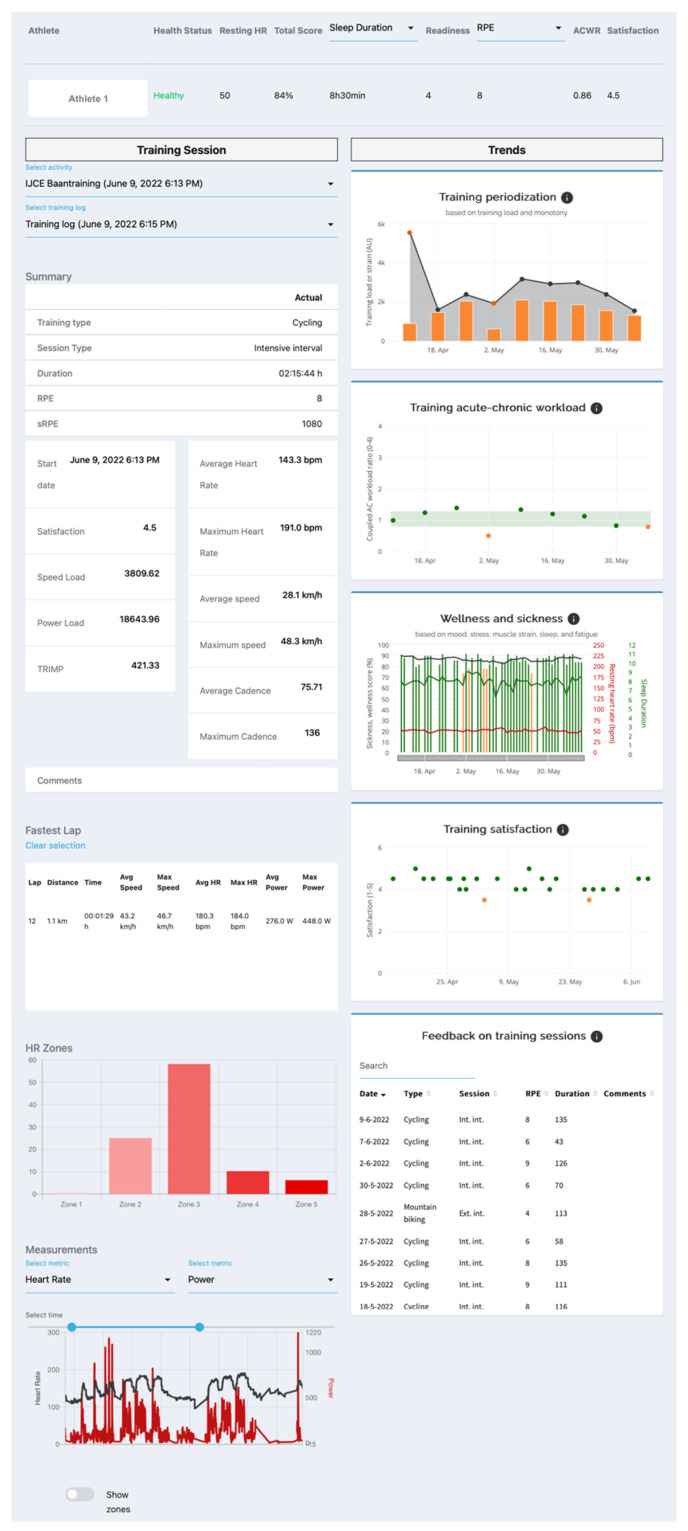
Display of the minimum viable product: Coach in Control dashboard. Training and Trends page. The Coach in Control dashboard is displayed as integrated in the Sport Data Valley platform [23]. Copyright 2022 Sport Data Valley.

The Agile Scrum design process [33] during the prototype phase took 20 weeks and resulted in the minimum viable product dashboard as displayed in Figure 4 and Figure 5. Health and Well-being page (Figure 4) was developed according to the mock-up, only the individual warnings based on well-being measures could not be implemented due to budgetary constraints. The health, well-being, and last training sessions’ measures are displayed in an overview display for the coach. The coach can choose which date is displayed. Figure 5 shows the Training and Trends page, which is divided into two panels. The left panel displays the detailed information from one activity performed by the chosen athlete and date. Data such as training type, RPE, session RPE (sRPE), training duration, training load, average velocity, power, and heart rate, etc., can be found there. The bottom part of the left panel shows information of the fastest recorded lap during the session, time in heart rate intensity zones and a figure of training measures displayed over training time. All the addressed measures in previous phases are implemented in this part of the dashboard. Trends are displayed in the right panel of the display (Figure 5) where weekly training load, Acute-to-Chronic Workload ratio (ACWR), and well-being, resting heart rate, and sleep duration trends of the chosen athlete in weekly summarised figures. All these data were high on the needs and functionality lists of the empathise and ideate phases.

We used a specification list based on the results of the ideate phase in the design. All specifications, 113 in total, were listed and prioritised by the product owner, and scored for complexity to implement by the scrum team. These priorities and complexity scores were used for decision making in the implementation of specifications in the coach dashboard. A few specifications set in earlier phases were adjusted for implementation in the minimum viable product dashboard according to these scores. To illustrate, the summary of the chosen training session is displayed different than was drawn in the mock-up. Development time, dashboard possibilities and issues, and renewed insights were the main reasons to adjust specifications. Additional functions to the mock-up were the feedback on a training session provided by the athlete, training data of the fastest lap during the training session, and calculating power and velocity training load. Weekly meetings with the scrum team and the product owner, where the priority and complexity of specifications were discussed, led to these adjustments in the development of the coach dashboard. Most important issues and adjustments are discussed here. These adjustments and other minor issues were registered in the backlog and specifications list.

For the coach, a maximum of 20 min analysis time was the most important need for working with a coach dashboard. Therefore, the dashboard should work quickly with short page load times. However, page load time when an athlete had a lot of data in the platform took too long. Coaches are most interested in the training-load data of the last couple of weeks; therefore, this issue was solved by only loading the last few weeks of training data of the selected athlete. Only when the coach selects a longer timeframe to see in the dashboard are older data of the athlete loaded into the dashboard.

Discussions with coaches showed that they were interested in comparing their intended training session design to the athletes experienced training load. This functionality was included on the Training and Trends page so that the coach can compare training modality, training type, training duration, and (s)RPE of the chosen training session and weekly training load. This comparison proved to be very useful and insightful. Written feedback by athletes on training sessions was not part of the specifications, but during the prototype phase coaches stated that this feedback was frequently used for their insights into the athletes’ experiences during and after training sessions. This feedback helped coaches interpret the monitored data, and therefore, the decisions for upcoming training programme and session design.

### 3.5. Test Phase—Practical Use of the Coach in Control Dashboard

Four coaches tested the feasibility of the minimum viable product: Coach in Control dashboard with a total of 31 athletes using the platform. In this phase, these feasibility tests were held after using the Coach in Control dashboard for 6–8 weeks, in order to give the coach the opportunity to use the functionalities of the dashboard in their daily practice. Interviews with coaches revealed that, once the connection between the coach and the athlete was made, the Coach in Control dashboard provided relevant information to the coach. Issues such as page load time were still addressed by coaches. “When the dashboard is loading it is not visible to me that it is loading, that annoys me” (C1). During this phase, we also learned that only instructing coaches how to use the Coach in Control dashboard is not enough. We had to put in effort and extensive instructions with the coach for them to use the dashboard as intended. “Especially when starting to use the platform, where can I find everything is a bit of a struggle for me” (C5). Every coach was therefore followed-up within the first 2 weeks of testing, and the coach was asked to show how they used the dashboard. This showed that all coaches needed additional help to use the full dashboard as intended.

After feasibility testing, the overall results were that the dashboard is useful in practice. Combining different data types (questionnaires, training log, and training activity sensor data) on one coach dashboard was seen as very helpful and time-saving to gain data-informed insights on training load. “Combining all activity data and the display over a longer time is super” (C6). Feedback for future improvements related to the navigation in and to the Coach in Control dashboard, which could be clearer, and to the direct comparison between the intended training load by the coach and experienced training load by the athlete, which could be improved. Strength of the adopted specifications was found in the scientific base of the specifications. For example, readiness, external load measures (e.g., velocity and power), TRIMP score, and ACWR are adopted in the dashboard.

## 4. Discussion

Designing a coach dashboard for coaches (i.e., Coach in Control dashboard), using the cooperative research design such as described in this paper, helped to gain deep insights into the needs and bottlenecks experienced by coaches in adopting such a dashboard. We will discuss advantages and disadvantages of using this approach in this chapter of the paper.

Combining scientific knowledge with insights from the survey and the interviews as performed in the empathise phase gave specific insights into the most relevant categories of items and topics in athlete monitoring. Reviewing all results gave a broad spectrum of initial design principles based on evidence to fit the coaches’ needs. Bottlenecks such as time, maximum 20 min’ analysis time for the coach, and using many data collection systems were addressed in developing the Coach in Control dashboard. When the dashboard was up and running between coach and athlete, the dashboard was able to help analyse training and provide insights into the trends. The Sport Data Valley platform [23] can help to collect and combine data from different systems as well. Starting from the theoretical training-load framework [6], our results provide more specific interpretation and measures to monitor the training load from a coach perspective. Using extensive cooperation methods including coaches in this phase as advised [34] is time-consuming but helped the decision making in design specifications in following design phases. Therefore, cooperating with coaches in this phase (and other phases as well) contributes to the use-in-practice of developed tools and monitoring [34]. Involving the end-user in design is well-known and practiced in design processes, but less in technological development and innovation in sports [20,24].

The define phase took place when COVID-19 restrictions were in place, which forced us to have the focus-group discussions online. Therefore, we had strict discussion rules enabling every participants’ opinion to be heard. Evaluation of these sessions showed that all participants felt free to give their opinion during the session. Input with the case and statements gave focus to the discussions and helped in defining the complete picture leading up to the ideate phase. Participants also noted that it was fun to participate and helped them understand other participants’ points of view as well, which helps in involving people in the design process and outcome [22]. A couple of participants mentioned that some statements were formulated sharply, and sometimes the discussion was more directed to the formulation of the statement rather than the content of the statement.

During the ideate phase in this study, all given input, scientific knowledge [5,13,15,31,35], and already-existing functionalities within the Sport Data Valley platform [23] were combined in the drawn mock-ups. As we were able to build upon the basis of an already-existing platform, we could use the infrastructure that was already implemented. Therefore, we could focus on combining data of daily questionnaires, training and activity monitoring data, and training log data in dashboard visuals and figures to provide combined insights for the coach that existing systems are not able to give [15,17,34]. Drawn mock-ups, therefore, built upon existing features but also added new features such as the Health and Well-being overview page.

Agile Scrum development [33] in the prototype phase was performed in a small team with only a software developer and a human-movement scientist. Risks involving working with a small team [36] were covered by consultations with coaches and IT professionals when necessary. Agile Scrum development with weekly meetings helped in keeping the development on track. The human-movement scientist was also involved in the prior phases and therefore had good insights into all addressed and discussed specifications. Therefore, questions of the scrum team could quickly be answered, keeping the development in a good flow. Even when the development was slow during a week, the sprints and meetings helped keep the development in focus.

In the test phase, interviews were used to collect qualitative data on the use-in-practice of the designed Coach in Control dashboard. During the development, we noticed that coaches used other functionalities of the Sport Data Valley platform as well. When asking about features and functionalities implemented in the Coach in Control dashboard, answers were provided based on existing Sport Data Valley dashboards or figures. Therefore, we chose interviews for data collection to make sure that coaches had the correct dashboard in mind. This intervention and helping the coach implement the dashboard in their daily practice working with athletes helped in recording relevant information in testing the dashboard.

Implemented features, scientific knowledge, and functionalities in the Coach in Control dashboard give the coach the possibility to make data-informed decisions on training planning and athlete development. An athlete monitoring cycle [37] and training-load measures [5,13,15,31,35] are adopted in the coach dashboard, and therefore, coaches can use monitored data to use the athlete monitoring cycle in their decision making [17,38]. To illustrate this, coaches can use the readiness score collected in the daily questionnaire for last-minute changes to the training session. External training load is collected based on the sport modality and used monitoring wearable sensors and systems [12,35]. Displayed data are training duration, distance, velocity (average, maximum, and load), power (average, maximum, and load), fastest lap data, and a graph displaying power and velocity over training duration. Internal training-load data implemented are heart rate (average, maximum, and TRIMP), heart rate zones, and a graph of heart rate over training duration. External and internal load data in the graph are combined so the coach can compare external to internal load. These features and functionalities help the coach adopt scientific knowledge on training monitoring [5,7,34]. Importantly, the dashboard also allows direct comparison between the intended training load by the coach and experienced training load by the athlete, which can have important implications for factors related to athlete development [14,39]. The fourth part of the athlete monitoring cycle is addressed with the athletes’ daily questionnaires, which provide insights on the health and well-being of the athletes. Quality and quantity of mood, stress, muscle soreness, sleep, fatigue, and resting heart rate [29,30,31] are collected and displayed daily and in trends in the Coach in Control dashboard. With these data, the coach can monitor athletes’ risk of non-functional overreaching and overtraining in response to the training programme [12]. With all these implemented features, the Coach in Control dashboard informs coaches, enabling them to make data-informed decisions for training and programming [34,38].

All coaches that participated in our study are coaching individual athletes in a cyclic sport such as speed skating, cycling, and running. Therefore, these results and interpretations cannot be immediately translated into designing a coach dashboard for other sports. Specific external load measures will, for example, differ between sports [40]. Internal load and subjective measures might be transferrable to other sports, but the needs and bottlenecks for monitoring that coaches experience can very well be different [34]. Incorporating coaches’ input in the complete design process [34], such as in this study, helped in gaining deeper understanding of their needs and use-in-practice of a coach dashboard. During the design phases, specifications and needs changed and shifted, leading to sharpening or adjustments of specifications, priorities, or functionalities. The designed Coach in Control dashboard is valued for addressing these changes leading up to a dashboard that has a good fit to the needs and use of coaches. Conducted interviews in the test phase learned that this will help in adopting the Coach in Control dashboard in the coaches’ daily work [22]. In particular, combining the different data types and used systems into a single dashboard and the time needed to make decisions based on these data are qualified as practical and relevant in the daily practice of coaches.

Added functionalities and training-load measures during the development of this coach dashboard are velocity and power training load. As adopted in the dashboard, both external training loads are calculated using the average velocity or power multiplied by training duration. Both calculations are a new feature trying to make external training load easier to understand for the coach as user. To give the coach “More information with fewer data”, as mentioned by one of the interviewed coaches (C1). Future research possibilities can be found in validating these velocity and power training loads and comparing them to internal training-load measures as well.

## 5. Conclusions

The newly co-created and developed Coach in Control dashboard demonstrated that coaches were able to use the dashboard in their daily training practice. Needs and bottlenecks addressed were the time needed for the coach to analyse training data and the athlete to provide the needed information daily. Moreover, the use of only one platform for all relevant training, health, and well-being data helps overcome another bottleneck for coaches. The Coach in Control dashboard, when used to the full extent, has the possibility to cover the complete athlete monitoring cycle [37]. Therefore, we conclude that the designed Coach in Control dashboard enables coaches’ decision making in training design and puts the ‘coach in control’ of the athletes’ development, with data based on the balance in training load, physical performance, mental health, and daily life factors at hand in one dashboard, and with dashboard functionalities that enable time-efficient work, provide easy-to-understand feedback, and fit the coaches’ context and workflow.

Limitations of this descriptive design study involve the following: (i) There is a need to identify insights into the effects of the use of this dashboard (and other dashboards as well) by coaches on their work and the performance development of their athletes; (ii) The Coach in Control dashboard design primarily focused on so-called cyclic sports (e.g., speed skating, running, cycling). How this dashboard can be used or changed to support coaches in other types of sports can be questioned; (iii) Due to the case study research design, only a selection of all possible training load, health, and well-being indicators were included in the newly developed dashboard. Future work in collaboration with more coaches can lead to changes in integrated parameters and data.

The main contribution of our work to the community is the used design process to develop this dashboard. Close collaboration with coaches, scientists, and IT professionals helped to bridge gaps between science, practice, and technology. This intensive co-creation process stimulates the engagement towards, and implementation of, the Coach in Control dashboard. Furthermore, the Coach in Control dashboard is fully functional and ready to use by any coach.

Future work using the Coach in Control dashboard can be found in: (i) Validating the implemented velocity load and power load calculations to existing methods and to the internal training loads; (ii) Identifying the relation of the use of the dashboard to results in athletes’ performance development and coaches workflow; and (iii) Exploring how using the dashboard can support communication between the coach and the athlete.

## Figures and Tables

**Table 1:** Survey results based on 38 respondents of the importance and use-in-practice of monitoring in training, testing, and competition. Based on 5-point Likert importance scale for ‘importance’ and regular use for ‘use in practice’. Average scores are displayed with standard deviation in between brackets. Asterisks shows statistically significant differences (*p* < 0.05, Friedman test) between the importance and use-in-practice.

Category	Importance	Use-in-Practice
Monitoring before training	3.9 (±0.5) *	2.9 (±0.4)
Monitoring after training	3.5 (±0.6) *	3.2 (±0.4)
Performance tests	3.4 (±0.4)	2.5 (±0.3)
Monitoring in competition	3.4 (±0.5) *	3.1 (±0.3)
Monitoring during training	3.3 (±0.5) *	2.6 (±0.6)

**Table 2:** Survey results based on 38 respondents of the importance given to available monitoring measures. Measures are displayed in ranks, the higher the score the more important the measure is. Average score is displayed in between brackets.

Before Training		During Training		After Training	
Injuries	(4.6)	Technical execution	(4.4)	Fun	(4.3)
Fatigue	(4.4)	Heart rate	(3.8)	Perceived load by the athlete	(3.8)
Recovery	(4.4)	Power	(3.7)	Training duration	(3.6)
Overtraining or overreaching	(4.4)	Repetition in strength training	(3.6)	Heart rate recovery	(3.5)
Health	(4.3)	Resistance in strength training	(3.5)	Distance covered	(2.3)
Fitness	(4.2)	Velocity	(3.5)		
Stress	(4.1)	Fluid balance	(3.3)		
Sleep	(3.9)	Number of accelerations (intervals)	(3.2)		
Performance behaviour	(3.8)	Barbell velocity in strength training	(2.8)		
Muscle feel	(3.7)	Lactate	(2.7)		
Mood	(3.7)	Temperature of the body	(2.7)		
‘Readiness to train’ survey	(3.6)	Weather	(2.4)		
Load estimation by the coach	(3.5)				
‘Readiness to train’ tests	(3.4)				
Growth	(3.0)				
Weight	(2.8)				

**Table 3:** Coach dashboard functionalities and feedback design principles based on the survey results.

Dashboard Functionalities		Feedback Design Principles	
Select myself which measures to see	(4.3)	Load of the individual athlete per week	(4.5)
Short navigation using few mouse clicks from an overview panel	(4.2)	Detailed feedback on individual data	(4.2)
Being able to design my own overview page	(4.2)	Load of the individual athlete per session	(4.1)
Collecting, processing, and analysing everything in one system	(4.2)	Feedback on training duration in zones	(4.1)
Sharing the training session with the athletes	(4.0)	Load of the individual athlete per day	(4.1)
Use monitoring equipment of my own choice	(4.0)	Global feedback on individual data	(3.9)
Being able to combine with existing systems	(4.0)	Feedback on how good the athlete followed the intended training design	(3.8)
Optimised for the laptop of desktop	(3.8)	Load of the individual athlete within a training session	(3.8)
Optimised for the smartphone	(3.6)	Feedback on average data over a training session	(3.6)
Optimised for the tablet	(3.5)	Load of the individual athlete per interval or set	(3.5)
Working in multiple systems	(2.7)	Global feedback on group data	(3.4)
		Feedback on lap times	(3.4)
		Detailed feedback on group data	(3.2)
		Load of the individual athlete per minute	(2.6)

**Table 4:** Ranking results of the four focus-group sessions combined. The ranking was performed according to the scoring order, where the Top 5 scored 1, 2, 3, 4, and 5 points. Statements in the optional bin scored a 6, and all scores 1 to 6 were approved and the corresponding statements will be considered as a required design principle. A score of 7 meant that the statement went to the discussion bin, and 8 and 9 were both rejected, with a score of 8 being a discussion and 9 being directly rejected. Therefore, the lower the score, the better the ranking.

Number	Statement	Ranking Score
1	In a maximum of 20 min, the coach should be able to use all individual training monitoring for the next training session	3.5
2	When athletes put in the system that they are injured, there will always follow a warning	3.8
3	With large differences within an individual the dashboard sends active warnings	4.3
4	Individual measures are used to provide insights into an ideal performance development	4.3
5	Integration with existing systems already used should be possible	4.5
6	The dashboard provides feedback based on individual heart rate and velocity zones as measured during performance tests	5.3
7	The coach can arrange the dashboard overview and reach the display in a few clicks	5.5
8	Based on the planned training session the dashboard calculates the predicted individual training load	5.7
9	Putting in data by the athlete must be performed with the smartphone in less than 5 min per day	5.7
10	The dashboard displays a comprehensive critical power profile including heart rate measures of the individual athlete	6.0
11	The dashboard shows detailed insights into individual measures	6.0
12	Feedback provided by the dashboard is presented with a confidence interval	6.3
13	Measures from training are being used to explain competition results	6.3
14	RPE as experienced by the athlete will be compared with the RPE prediction as provided by the coach	6.3
15	The dashboard calculates every measure relatively based on the individual athlete’s history	6.3
16	The dashboard must have the possibility to share the training programme with the athletes	6.8
17	The dashboard provides adjustments in training intensity per individual athlete	7.0
18	For EUR 150, I expect, besides the use of the dashboard, support in use as well	7.0
19	The dashboard should mainly be presented in overview on a desktop or laptop	7.3
20	The dashboard provides a single measure of training performance in relation to the athletes’ history based on training monitoring measures	7.3
21	The dashboard displays the training load measures separately so the coach can calculate the training load	7.3
22	The dashboard displays only the total readiness score per individual athlete	7.3
23	The dashboard calculates the total training load and displays this in one value	7.3
24	The dashboard sends the coach warnings when large differences between individuals are present	7.5
25	Performance behaviour data will be saved in the dashboard without consideration in analyses	7.7
26	The dashboard provides adjustments in training duration per individual athlete	8.0
27	Finish times and competition rankings will be saved in the dashboard without insights over time	8.3
28	The longest period of individual training load displayed in the dashboard is per week	8.5
29	The dashboard predicts competition performances based on individual measures	8.5
30	The coach gives a grade in the dashboard for the technical execution during training	8.7

**Table 5:** Scored items (functionalities, content, design, overview, simplicity, and insights) as given in showcase and discussion of the second mock-up of the coach dashboard. A 5-point rewarding scale was used. The first mock-up was only discussed on these items but not scored. Average score is displayed between brackets.

Mock-Up (Version 2)	Health and Well-Being Page	Training and Trends Page
Functionalities	4.0 (±0.6)	4.0 (±0.0)
Content	4.2 (±0.4)	4.2 (±0.4)
Design	3.8 (±0.7)	3.6 (±0.5)
Overview	4.0 (±0.6)	3.6 (±0.8)
Simplicity	3.2 (±0.7)	3.0 (±0.6)
Insights	3.8 (±0.7)	4.0 (±0.6)

**Figure A1:**
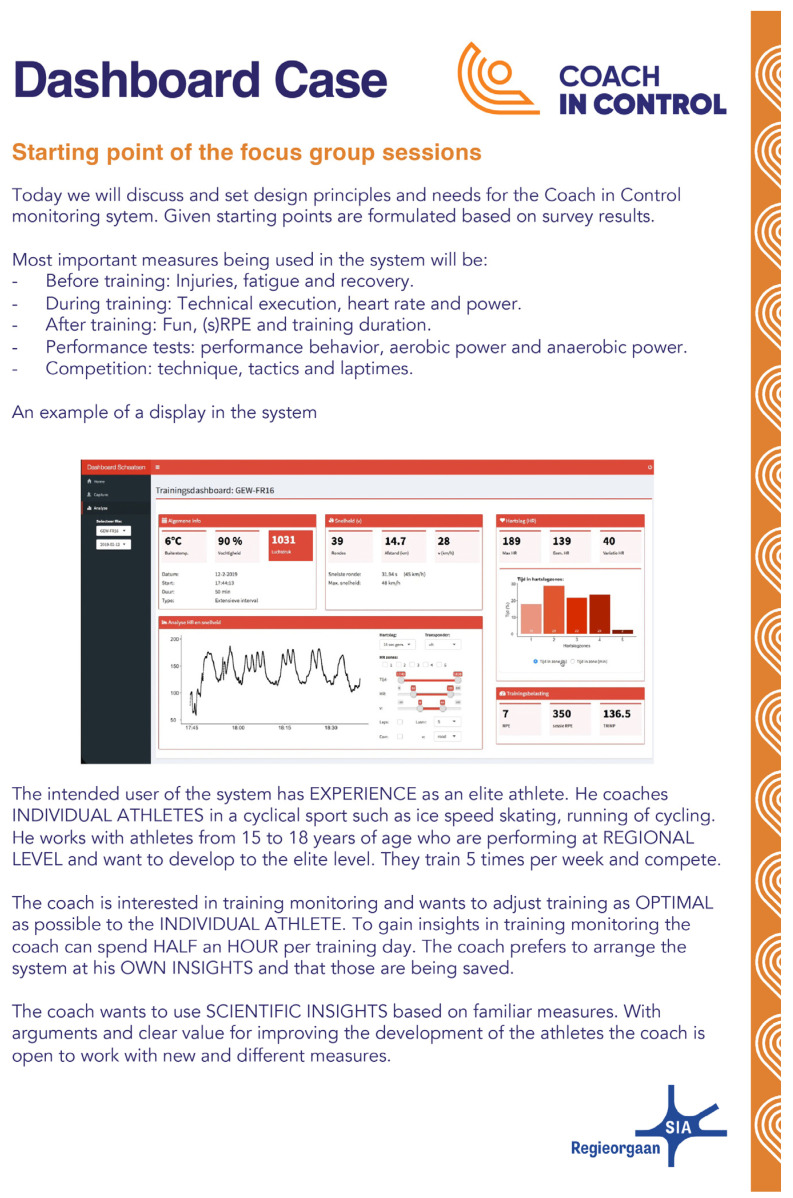
Used case during the focus-group sessions in the define phase.

**Figure A2:**
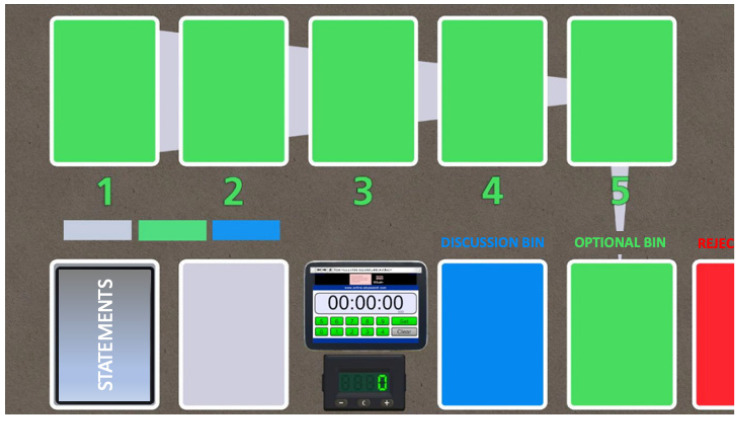
Visual of the digital COMMONS board, based on Arts et al. [28]. The green places at the top are the top-1 (highest prioritised statement) to top-5 (fifth prioritised statement) places, with the optional bin at the bottom. The red place is the rejected bin, which was only half-visible on the screen during the focus group as well. In the middle, a timer and counter are displayed. For every statement, there was a voting round in which participants chose to approve (green card), rejected (red card), or had no specific preference (blue card). When all participants voted green and/or blue, the statement was approved and had to be placed and prioritised in the already-placed statements on the tangible board. All red and blue meant the statement went to the rejected bin. Any combination of red, green, and/or blue led to a 3-min discussion between the participants. After the discussion, all participants gave their second and final vote to place the statement. When there was still a red, green, and blue combination, the card moved to the discussion bin. The statement could also go to the approved section on the board or rejected bin as well. This discussion approach was based on the consent decision-making method [41]. Copyright 2022 Fontys School of Applied Sciences.

## Data Availability

Not applicable.

## Appendix B

**Table A1:** Overview of all 30 statements used in the focus group sessions during the Define phase.

Number	Statement
1	In a maximum of 20 min, the coach should be able to use all individual training monitoring for the next training session
2	When athletes put in the system that they are injured, there will always follow a warning
3	With large differences within an individual the dashboard sends active warnings
4	Individual measures are used to provide insights into an ideal performance development
5	Integration with existing systems already used should be possible
6	The dashboard provides feedback based on individual heart rate and velocity zones as measured during performance tests
7	The coach can arrange the dashboard overview and reach the display in a few clicks
8	Based on the planned training session the dashboard calculates the predicted individual training load
9	Putting in data by the athlete must be performed with the smartphone in less than 5 min per day
10	The dashboard displays a comprehensive critical power profile including heart rate measures of the individual athlete
11	The dashboard shows detailed insights into individual measures
12	Feedback provided by the dashboard is presented with a confidence interval
13	Measures from training are being used to explain competition results
14	RPE as experienced by the athlete will be compared with the RPE prediction as provided by the coach
15	The dashboard calculates every measure relatively based on the individual athlete’s history
16	The dashboard must have the possibility to share the training programme with the athletes
17	The dashboard provides adjustments in training intensity per individual athlete
18	For EUR 150, I expect, besides the use of the dashboard, support in use as well
19	The dashboard should mainly be presented in overview on a desktop or laptop
20	The dashboard provides a single measure of training performance in relation to the athletes’ history based on training monitoring measures
21	The dashboard displays the training load measures separately so the coach can calculate the training load
22	The dashboard displays only the total readiness score per individual athlete
23	The dashboard calculates the total training load and displays this in one value
24	The dashboard sends the coach warnings when large differences between individuals are present
25	Performance behaviour data will be saved in the dashboard without consideration in analyses
26	The dashboard provides adjustments in training duration per individual athlete
27	Finish times and competition rankings will be saved in the dashboard without insights over time
28	The longest period of individual training load displayed in the dashboard is per week
29	The dashboard predicts competition performances based on individual measures
30	The coach gives a grade in the dashboard for the technical execution during training
